# The antibacterial activity of methanolic *Anacyclus pyrethrum* and *Pistacia lentiscus L.* extract on *Escherichia coli*

**Published:** 2016-12

**Authors:** Noushin Jalayer-Naderi, Mohammad Niakan, Elham khodadadi, Maryam Mohamadi-Motlagh

**Affiliations:** 1 Department of Oral and Maxillofacial Pathology, Faculty of Dentistry, Shahed University, Tehran, Iran; 2 Department Microbiology, Faculty of Medicine, Shahed University, Tehran, Iran; 3 Graduate Student, Faculty of Dentistry, Shahed University, Tehran, Iran

**Keywords:** Antibacterial activity, *Anacyclus pyrethrum*, *Pistacia lentiscus L*., *Escherichia coli*

## Abstract

**Background and Objectives:**

Antibiotic therapy is the main choice in treatment of *Escherichia coli* induced infections. Using herbal medication is an alternative choice in treatment of diseases. The aim of this study was to determine the antibacterial activity of two traditionally used herbs in Iranian medicine, *Anacyclus pyrethrum* and *Pistacia lentiscus L.,* on *Escherichia coli.*

**Materials and Methods:**

The antibacterial effect of methanolic extract of *Anacyclus pyrethrum* and *Pistacia lentiscus L.* were examined in disk diffusion and skipped wells methods by measuring the diameter of inhibition zones around wells containing different concentrations of extracts from (10–1000 mg/ml) using standard broth macrodilution, method the MIC and MBC were defined.

**Results:**

The methanolic extract of *Anacyclus pyrethrum* from 300 to 1000 mg/ml and the methanolic extract of *Pistacia lentiscus L.* from 30 to 1000 mg/ml showed antibacterial activity on *Escherichia coli.* The MIC of *Anacyclus pyrethrum* and *Pistacia lentiscus L.* methanolic based extract were 800 and 1000 mg/ml, respectively. The MBC was achieved at 800 mg/ml for methanolic extract of *Anacyclus pyrethrum* and *Pistacia lentiscus L.*

**Conclusion:**

The methanolic extract of *Anacyclus pyrethrum* and *Pistacia lentiscus L.* have antibacterial effect on *Escherichia coli* bacteria. This activity is dose-dependent.

## INTRODUCTION

*Escherichia coli* is a facultative Gram negative bacterium that is generally found in the lower intestine (1). The virulent strains of *E. coli* cause gastroenteritis and infections of urinary tract. In addition to fluid and electrolytes replacement, the antibiotic therapy is the main choice in treatment of diseases caused by this organism (2–3).

Antibiotic therapy is facing with different problems ranging from hypersensivity to bacterial resistance. Herbal medication is an alternative choice in treatment of different diseases. The native herbal - based medicine are easy available and non-expensive. The effectiveness of some native herbal - based medicine on *E. coli* has been shown in Africa, England and China (4–7).

*Anacyclus pyrethrum* (locally known as akarkara) and *Pistacia lentiscus L.* (locally named mastaki) are traditionally used herbs in Iranian medicine by physicians such as Avicenna (8). *Anacyclus pyrethrum* (pellitory) from *Anacyclus* genus is a native plant of India and Arabic countries. It has different therapeutic effects such as antmicrobial, analgesic and antioxidant activitivities (9–10). It has been shown that *Anacyclus pyrethrum* has antimicrobial effects on *Candida albicans, Staphylococcus aureus* and strong larvicidal activity against malaria (11–12).

*Pistacia lentiscus L.* is a native herb of Africa and Mediterranean countries with antimicrobial, anti-fungal, antioxidant and anti-inflammatory activities (13–14). It has good effect on gastrointestinal diseases due to its anti *Helicobacter pylori* activity (14–15).

The aim of this study was to determine the antibacterial activity of *Anacyclus pyrethrum* and *Pistacia lentiscus L.* on *E. coli.* Based on our knowledge this is the first study on the antibacterial activity of methanolic extract of *Anacyclus pyrethrum* and *Pistacia lentiscus L.* on this organism

## MATERIALS AND METHODS

The antibacterial activites of methalonic extracts of *Anacyclus pyrethrum* and *Pistacia lentiscus L.* on *E. coli* were examined using well diffusion method. The *Anacyclus pyrethrum* and *Pistacia lentiscus L.* were purchased from traditional pharmacies in Shiraz, Iran. The samples were verified by the Department of Pharmacognosy, School of Pharmacy, Shahid Beheshti University of Medical Sciences, Tehran, Iran.

### Extraction

200ml of 95% methanol was added to 200g of the chopped, powdered roots of *Anacyclus pyrethrum* and *Pistacia lentiscus L.* in a sterile flask. The mixture left for 24 hours at room temperature and filtered with No.1 filter paper (Whatman Co Germany.) with 150μm diameter for liquor filtration. The extract was dried in Water bath at 70 C° for one week. Dried powdered extract was kept at 4°C at tightly closed vial. Different concentrations from 10 mg/ml to 1200 mg/ml in distilled water were prepared from stock solution (16).

### Organism

*Escherichia coli* (ATCC 25922) was obtained from the bacterial collection of Department of Microbiology, Medical Faculty, Shahed University.

### Disk diffusion test

A swab of bacterial suspension was streaked on Muller Hinton agar plates (Liofilchem Company, Italy).

The columns adjusted to 1.5 × 10^8^ bacterial/ml and used sterile blank disks of extract dilution (McFarland 0.5 turbidity standard). After overnight 37°C aerobically condition incubation, the zones of inhibition were measured by using an Antibiotic disk Zone Reader after 24 h.

### Minimum Inhibitory Concentration (MIC) and Minimum Bactericidal Concentration (MBC)

The MIC was measured by broth Macro dilution test (tube Dilution) method updated by Clinical and Laboratory Standards Institute (17). The lowest concentration of extract that inhibited the visible growth of organism was recorded as MIC. 10 μl of bacterial suspensions with turbidity of McFarland 0.5 were added to test tubes. 1ml of incubated solution with determined MIC were cultured and kept incubated at 37°C for 24 h. The antimicrobial extracts concentration that show inhibition (>MIC) may have killed the bacterium. Subculture plate less than 0.1% of the initial inoculum, first concentration without bacteria growth was considered as the MBC.

## RESULTS

### 

#### Anacyclus pyrethrum

The methanolic extract of *Anacyclus pyretprum* had not inhibitory effect on *E. coli* at 10 to 200 mg/ml concentrations. From 300 to 1000 mg/ml the inhibition zone was noted.

Table 1. shows the inhibition zone diameters in different concentrations of methanolic extract of *Anacyclus pyrethrum* on *E. coli.*

**Table 1.** The inhibition zone diameter of methanolic extract of *Anacyclus Pyrethrum* and *Pistacia lentiscus L.* on *Escherichia coli*

**Extract**	**10 mg/3ml**	**20 mg/ml**	**30 mg/ml**	**40 mg/ml**	**50 mg/ml**	**100 mg/ml**	**300 mg/ml**	**400 mg/ml**	**600 mg/ml**	**800 mg/ml**	**1000 mg/ml**
***Anacyclus Pyrethrum***	0	0	0	0	0	0	15 mm	15 mm	17 mm	20 mm	23 mm
***Pistacia lentiscus L.***	0	0	8 mm	12 mm	15 mm	18 mm	19 mm	19 mm	12 mm	15 mm	17 mm

**Table 2.** Antimicrobial activity of *Anacyclus pyrethrum* and *Pistacia lentiscus L.* methanolic extract on *Escherichia coli* by determining MIC

**Extract**	**10 mg/ml**	**20 mg/ml**	**30 mg/ml**	**40 mg/ml**	**50 mg/ml**	**100 mg/ml**	**300 mg/ml**	**400 mg/ml**	**600 mg/ml**	**800 mg/ml**	**1000 mg/ml**
***Anacyclus pyrethrum***	+	+	+	+	+	+	+	+	+	MIC-	MIC-
***Pistacia lentiscus L.***	+	+	+	+	+	+	+	+	+	+	MIC-

## DISCUSSION

The study shows methanolic extract of *Anacyclus pyrethrum* and *Pistacia lentiscus L.* have antibacterial effect on *E. coli.* This activity is dose-dependent.

Some previous researches have shown the effectiveness of native derived herbal - based medicine on *E. coli* such as the mixture of combining *Agathosma crenulata, Dodonaea viscosa* and *Eucalyptus globulus* from Africa and *Potentilla reptans L.* from Anglo-Saxon native herbs (4–5).

*Parkia biglobosa(Jacq.) Benth, Ageratum conyzoides Linn.* From African countries and *M. yunnanensis, S. sinensis, G. morella, E. daneillii, M. squamulata, S. arborescens* and *B. hancei* from native Chinese herbal medicine have also antibacterial effect on *E. coli* (6–7).

The study shows the methanolic extract of *Anacyclus pyrethrum* from 300 to 1000 mg/ml had inhibitory effect on *E. coli.* Based on our knowledge this is the first study on the antibacterial activity of methanolic extract of *Anacyclus pyrethrum* on *E. coli.*

Selles et al. showed that the essential oil from Algerian *Anacyclus pyrethrum L.* has activity against *Candida albicans* and *Staphylococcus aureus.* Oxygenated sesquiterpenes was the main effective substance (12).

Studies on biologically active substance of *Anacyclus pyrethrum* and its antibacterial effects are very rare. It has not been elucidated that which active component or either biologic mechanism of *Anacyclus pyrethrum* has antibacterial activity on *E. coli.*

The antibacterial activity of *Pistacia lentiscus L.* was studied more than *Anacyclus pyrethrum.* Some studies on this field were focused on oral cavity bacteria (18–20). It has been reported that *Pistacia lentiscus L.* has antibacterial effecton *Staphylococcus aureus, Bacillus subtilis, Prevotella melaninogenica* and *Klebsiella pneumoniae* (21–24).

The alpha-Pinene, beta-myrcene, beta-pinene, limonene, and beta-caryophyllene are major antibacterial components of essential oil and gum of *Pistacia lentiscusL. Escherichia coli, Staphylococcus aureus,* and *Bacillus subtilis* have different sensitivity to these compounds (21).

It seems that different components of *Pistacia lentiscus L.* have different effects on different bacteria. Mharti et al. demonstrated that germanicol, thunbergol, himachalene, trans-squalene, terpinyl propionate, 3,3-dimenthol and cadina-1.4-diene derived from essential oil of the leaves of *Pistacia lentiscus L.* have antibacterial effect on *Klebsiella pneumoniae*, but not on *Pseudomonas aeruginosa* (23).

The present study shows that methanolic extract of *Anacyclus pyrethrum* and *Pistacia lentiscus L.* have antibacterial effect on *E. coli.* More researches on identifying the effective components of *Anacyclus pyrethrum* and *Pistacia lentiscus L*. on *Escherichia coli* are necessary.

In conclusion, the methanolic extract of *Anacyclus pyrethrum* and *Pistacia lentiscus L.* have antibacterial effect on *Escherichia coli* bacteria. This activity is dose-dependent.
